# Impacts of impervious surface expansion on soil organic carbon – a spatially explicit study

**DOI:** 10.1038/srep17905

**Published:** 2015-12-08

**Authors:** Yan Yan, Wenhui Kuang, Chi Zhang, Chunbo Chen

**Affiliations:** 1 State Key Laboratory of Desert and Oasis Ecology, Xinjiang Institute of Ecology and Geography, Chinese Academy of Sciences, Urumqi 830011, Xinjiang, China; 2 University of Chinese Academy of Sciences, Beijing, 100049, China; 3 Institute of Geographic Sciences and Natural Resources Research, Chinese Academy of Sciences, Beijing, 100101, China; 4 Global Institute of Sustainability, Arizona State University, AZ, United States

## Abstract

The rapid expansion of impervious surface areas (ISA) threatens soil organic carbon (SOC) pools in urbanized areas globally. The paucity of field observations on SOC under ISA (SOC_ISA_), especially in dryland areas has limited our ability to assess the ecological impacts of ISA expansion. Based on systematically measured SOC_ISA_ (0–80 cm depth) of a dryland city, and land-use and land-cover change data derived from remotely sensed data, we investigated the magnitude and vertical/horizontal patterns of SOC_ISA_ and mapped the impact of ISA expansion on SOC storage. The mean SOC_ISA_ in the city was 5.36 ± 0.51 kg C m^−2^, lower than that observed in humid cities but much higher than that assumed in many regional carbon assessments. SOC_ISA_ decreased linearly as the soil depth or the horizontal distance from the open area increased. SOC_ISA_ accounted for over half of the city’s SOC stock, which decreased by 16% (primarily in the converted croplands) because of ISA expansion from 1990 to 2010. The impacts of the ISA expansion varied spatially, depending on the land- use and converted land-cover type.

Urban expansion has converted large areas of cultivated lands, grasslands, and forests to built-up areas dominated by impervious surfaces (ISA), including roads, roofs, and parking lots^1^,^2^,^3^,^4^. Impervious surfaces cover approximately 1.3% and 9% of the land areas in the United States of America (USA) and Europe, respectively^5^,^6^. Globally, China has the largest area of impervious surfaces^7^, which covers approximately 63% of its rapidly expanding urban lands^8^. From 2000 to 2008, China’s urban ISA increased by 53.3%^8^. The installation of impervious surfaces usually involves the removal of vegetation and topsoil, and leads to soil compaction and sealing^5^. Soil sealing may effectively limit the exchange of water, gas, and biomass between urban soils and the atmosphere^9^, and significantly alter the biogeochemical characteristics of the converted soil^10^,^11^. Quantifying urban SOC storage is important for assessing carbon budgets within urban ecosystems^12^ and evaluating the impacts of urbanization on the global carbon cycle^4^. Pataki *et al.*^13^ emphasized that assessments of the urban SOC stock might be highly sensitive to whether counting is performed in SOC_ISA_. However, our knowledge about the physical (e.g., bulk density, BD) and biogeochemical (e.g., SOC) characteristics of the soils underneath impervious surfaces is limited because of the paucity of data^4^. As a result, previous carbon cycle studies had to rely on over-simplified assumptions to assess the SOC stock beneath ISAs. Many studies assumed that urban land conversion would remove all soil organic matter, resulting in low soil organic carbon density (SOCD_ISA_) for impervious surfaces^14^,^15^,^16^,^17^. Some studies set constant values for SOCD_ISA_ (e.g., SOCD_ISA_≡1.0 kg C m^−2^ in two European urban studies^18^,^19^ and SOCD_ISA_≡3.3 kg C m^−2^ in two USA urban studies^20^,^21^, both at 0–100 cm depth). Other studies used the SOCD of the adjacent pervious surface areas (SOCD_PSA_) to approximate the SOCD_ISA_^22^,^23^. Different assumptions may lead to significantly different conclusions on urbanization effects^24^, and thus should be evaluated with field observations.

However, field investigations on SOCD_ISA_ are difficult to conduct. All of the previous field studies have been conducted in humid or semi-humid regions^12^,^25^,^26^, while the SOCD_ISA_ in the cities in dryland climates, which accounts for more than one-third of the global land area and support more than 20% of human population^25^, is still unknown. With low precipitation and high evaporation rates, the soils in dryland regions have relatively low SOCD in comparison to humid ecosystems, due to lower carbon input from the water-stressed vegetation^25^. Meanwhile, intensive human disturbances and/or management actions may modify the SOCD in urban areas, altering the SOCD differences among cities under different climate regimes^26^. To understand how urban soils are influenced by climate and the associated environmental constraints, it is necessary to investigate the SOCD_ISA_ in dryland regions. Such knowledge is especially important to our understanding of the spatial variations in SOCD_ISA_ at the continental to global scale. At the city scale, the complex urban landscape and land-use change history might result in strong spatial heterogeneity in urban SOC distribution^27^. Until now, the spatial distribution of SOC in urbanized areas and its response to ISA expansion are unclear.

It is noteworthy that a fraction of SOC in dryland soils is located deep within the soil profile^28^. Knowledge about the vertical distribution of SOCD is important for assessing the impact of urbanization on dryland SOC balance^29^. However, the few studies on SOCD_ISA_^12^,^30^,^31^ provide adequate information about the spatial (vertical and horizontal) patterns, which could provide evidence of the impact of human disturbances and surface sealing on soil biogeochemistry. Although Edmondson *et al.*^12^ investigated the correlation between soil depth and SOCD_ISA_, uncertainties still exist because their sampling depths varied from plot to plot. Other SOCD_ISA_ studies mainly focused on the topsoil^30^,^31^. Furthermore, previous studies have not investigated the horizontal pattern of SOCD_ISA_. It has been suggested that soil sealing may radically alter the biogeochemical processes in soils^5^,^9^. Analysis on the horizontal gradient of SOCD_ISA_ from the center of ISA (where mass exchanges between the soil and the atmosphere are minimal) and the edge of ISA (where mass exchanges between the ISA soil and the adjacent pervious surface area (PSA) exist) might provide evidence of the sealing effects on soil’s biological activities.

Based on systematically measuring SOC_ISA_ and the land-use and land-cover change data derived from remote sensing of a dryland city, we quantified the SOC_ISA_ and investigated vertical and horizontal patterns of SOC_ISA_, and further mapped the impact of ISA expansion on the SOC stock of a city. Our objectives were to (1) measure the SOCD of the impervious surfaces in a dryland city and compare the results with previous findings in humid cities, (2) investigate the vertical and horizontal variations of SOCD_ISA_, and (3) assess the impacts of ISA expansion on the SOC stock of a dryland city, and reveal the spatial distribution of soil carbon sources/sinks. To our knowledge, this is the first time that the spatial pattern of the SOCD_ISA_ in a city has been mapped and the impact of ISA expansion on the SOC distribution quantified.

## Results

### Spatial patterns of BD and SOC in urban soils

According to paired t-tests, the BD_ISA_ (1.62 ± 0.04 g cm^−3^) was significantly (2-tailed, *p = *0.0096) higher (by approximately 8%) than the BD_PSA_ (1.49 ± 0.02 g cm^−3^), whereas the soil organic carbon content beneath ISA (SOCC_ISA_) (3.55 ± 0.36 g kg^−1^) was significantly (2-tailed, *p* = 0.010) lower than the SOCC_PSA_ (5.47 ± 0.58 g kg^−1^) (Table 1). The SOCD_ISA_ (5.36 ± 0.51 kg C m^−2^) was only 66% of the SOCD_PSA_. The SOCD_ISA_ was also lower than the mean SOCD (6.14 ± 0.88 kg C m^−2^) of the rural soils (SOCD_rural_) in the region^32^. The SOCDs of the different land covers are compared in Supplementary Figure S1.

**Figure 1:**
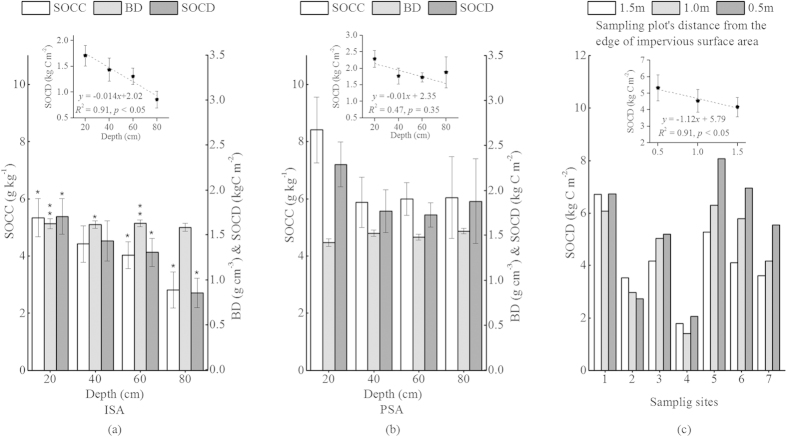
Comparison of vertical distribution of soil organic carbon content, soil bulk density and soil organic carbon density between (**a**) urban impervious surface and (**b**) pervious surface at each layer (** and * above the error bars indicate significant differences between the soil_ISA_ and soil_PSA_ at the 0.01, 0.05 levels, respectively), and (**c**) the horizontal gradient of soil organic carbon from the edge of the impervious surface.

Under the impervious surfaces, the SOCC and BD of the upper soil layers (0–20 cm and 20–40 cm) were higher than that of the lower soil layers (40–60 cm and 60–80 cm). The negative relationship between the SOCD_ISA_ (kg C m^−2^) of a soil layer and its depth (cm) was described by a significant linear function (*R*^2^ = 0.91; *k = *-0.014, *p* < 0.05) (Fig. 1a). We did not find a significant vertical trend in the SOCD_PSA_ (Fig. 1b). The SOC_ISA_ was significantly lower (2-tailed, p < 0.01) than the SOC_PSA_ at most soil layers except for the 20–40cm layer, while the BD_ISA_ was significantly higher than the BD_PSA_ at all soil layers above 60 cm (Fig. 1a,b). Moreover, the SOCD_ISA_ decreased linearly (*R*^*2*^ = 0.91, *k = *−1.12, *p* < 0.05), while the BD_ISA_ increased (*R*^*2*^ = 0.98, *k* = 0.003, *p* < 0.05) from the edge of the impervious surface to the center (Fig. 1c).

### Impacts of ISA expansion on the SOC stock of Urumqi

**Figure 2:**
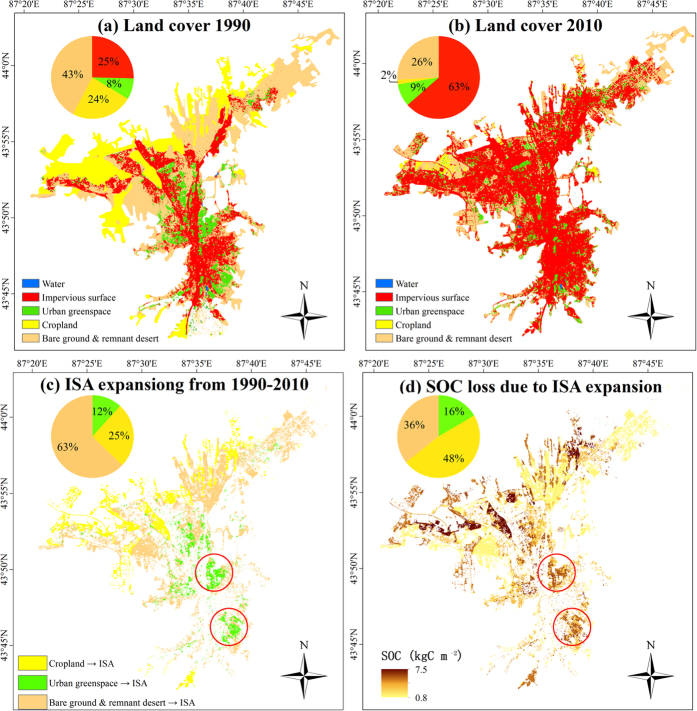
The land-cover types (impervious surface, urban green spaces, bare ground, and cropland) of Urumqi in 1990 (**a**) and 2010 (**b**), the expansion of impervious surface area (ISA) from 1990–2010 (**c**), and the carbon loss caused by ISA expansion (**d**). This map was generated by the one of the authors (Yan Yan) by using ArcMap (version 10.2).

ISA expansion was the dominant land-cover change in Urumqi during the recent two decades (Fig. 2a–c). The coverage of ISA in the Urumqi metropolitan area increased from 25% in 1990 to 63% in 2010. During this twenty-year period, approximately 137 km^2^ of land were converted to impervious surfaces, whereas croplands and bare ground/remnant deserts shrank by 79 km^2^ and 59 km^2^, respectively. According to its area and mean SOCD (Supplementary Figure S1), the SOC_ISA_ pool in the Urumqi metropolitan area was approximately 1.23 Tg C in 2010, accounting for over 57% of the total SOC stock in the city.

Further analysis of the spatial pattern of land-cover change revealed that the impervious surface mostly sprawled northward displacing remnant desert/bare ground and cropland (Fig. 2c). Urban infill was found in the south and mid-region, where four large patches of urban green space had been converted to impervious surfaces. From 1990–2010, approximately 85.8 km^2^ of remnant deserts/bare ground, 34.74 km^2^ of cropland, and 16.07 km^2^ of urban green space were converted to impervious surfaces in the Urumqi metropolitan area. The city lost 0.77 Tg SOC (or 16% of its SOC stock in 1990) as a result of ISA expansion. It is worth noting that 48% of the carbon loss was caused by the conversion of cropland to ISA (Fig. 2d) although it only accounted for 25% of the ISA expansion (Fig. 2c).

## Discussion

### Evaluation of the SOCD_ISA_ assumptions and their impacts on urban carbon assessments

Most urban soil studies have been conducted in PSA^9^,^20^ (supplementary Table S1). The SOC_ISA_ was either approximated with the adjacent SOC_PSA_^22^,^23^ or estimated from the mean SOC of pervious sites^20^. However, our study showed that the ISA and PSA differed significantly in SOC and BD (Table 1), especially in the topsoil (0–20 cm in our study) where the SOCD_ISA_ was only 75% of the adjacent SOC_PSA_. Similarly, a study in New York City found the topsoil (0–15cm) organic carbon under ISA was 66% lower than that of the adjacent PSA soil^30^. Further, we showed that the ISA and PSA had different vertical SOC and BD distribution patterns, and the SOCD_ISA_ was significantly lower than the SOCD_PSA_ in most soil layers (Fig. 1). These findings indicate that PSA should not be used as a proxy for soil under ISA.

Accordingly, the patterns and theories derived based on PSA studies may not be applicable to ISA. For example, the urban ecosystem convergence theory suggests cities in different climate zones could have similar SOCDs as the result of intensive human managements, even if their native SOCDs differ significantly due to the climatic constrains^26^. Wei *et al.*^33^ suggested the disturbed soils under ISA might reach an “equilibrium SOCD_ISA_ value” that does not vary significantly among cities in different climate zones, and the SOC storage in impervious soils at continental or global scales could be estimated based on this “equilibrium SOCD_ISA_ value”. Our data showed the SOCD_PSA_ in the dryland city (Urumqi) was close to the reported SOCD_PSA_ in humid cities (supplementary Table S1), thus supported the urban ecosystem convergence theory^34^ in PSAs. The SOCD_ISA_ in the dryland city, however, was lower than that in the humid cities (Table 2), suggesting an “equilibrium SOCD_ISA_ value”^34^ may not exist. This finding indicated possible climate (humidity or precipitation) control over the SOCD_ISA_, and called into question the use of constant SOCD_ISA_ values across large areas in carbon budget assessments^18^,^19^,^20^,^21^.

It is noteworthy that most carbon budget assessments assumed extremely low SOCD_ISA_ values (1.0 kg C m^−2^ to 3.3 kg C m^−2^)^18^,^19^,^20^,^21^, which is even lower than the SOCD_ISA_ (5.36 ± 0.51 kg C m^−2^) of the dryland city of Urumqi. Such assumptions could lead to significant underestimates of urban carbon budget at national to global scales. For example, a conservative re-assessment of SOC_ISA_ in the USA based on our observations in a dryland city indicates that Churkina *et al.*^21^ might have underestimated the SOC stock in the developed areas of USA by 0.3 Pg, equivalent to the forest carbon stock in Kentucky^35^. Urban SOC_ISA_ assessments should be based on field observations rather than untested assumptions.

### Spatial patterns of SOC_ISA_ and the implications for the underlying mechanisms

Unlike previous studies that primarily focused on topsoil^30^,^31^, we systematically examined the vertical and horizontal distributions of the SOC_ISA_ (Fig. 1). Such information may provide information about the influence of impervious surface on soil structure and biogeochemical processes^12^. We found urban SOCD_ISA_ declined linearly with depth, similar to the patterns of rural soils (supplementary Figure S2), but not so with SOCD_PSA_ that had no vertical trend due to intensive human disturbances^36^,^37^,^38^,^39^. This finding indicates that the construction of ISA may not severely disrupt the subsoil layers, and the pre-urban vertical pattern of SOCD was largely maintained, possibly because the installation of impervious surface protected the soil from frequent disturbances and slowed the SOC decomposition and leaching processes^30^,^31^. However, the physical soil structure was changed significantly under the ISA. The BD_ISA_ (1.62 g cm^−3^) was significantly higher than the BD_PSA_ (1.49 g cm^−3^) and the BD of rural soils (1.28 g cm^−3^)^40^. Unlike the rural soil profile whose BD increases with depth^41^, the mean BD_ISA_ of the upper soil layers (0–40 cm) was higher than that of the lower soil layers (40–80 cm). Furthermore, the BD_ISA_ increased significantly from the edge of the impervious surface to the center, leading to high BD (1.67 g cm^−3^) in the center of the ISA. These findings indicate the ISA construction could significantly increase the soil compactness to the level (~1.69 g cm^−3^) that can reduce the root growth by 50%^42^.

Our analysis of the horizontal pattern of SOCD_ISA_ provided evidence of how soil sealing may affect biogeochemical processes. Because the impervious surfaces prevent vertical carbon influx to the soil, the SOC dynamic under ISA is mainly determined by the balance between two processes^30^,^31^: (1) the horizontal infiltration of carbon from the PSA, which could lead to a higher SOCD at the edge of the ISA, and (2) the SOC decomposition that was controlled by environmental factors such as soil oxygen availability. The closer to the PSA (or the edges of ISA) the soil oxygen concentration is higher, possibly leading to a higher SOC decomposition rate and a lower SOCD at the edge of ISA. The SOCD_ISA_ distribution pattern predicted by the oxygen-limitation theory, however, did not match our finding that indicated a declining SOCD gradient from the edge to the center of an impervious surface (Fig. 1c). This horizontal pattern of SOCD_ISA_ could be caused by the horizontal infiltration of carbon from the PSA or might be related to other environmental controls on soil decomposition (e.g., the horizontal gradient of soil temperature). More field measurements and experiments are required to improve our understanding of the underlying mechanisms.

### Urbanization and carbon management

The impacts of urbanization on ecosystem carbon balance have gained attention from policymakers as well as scientists^4^,^43^,^44^. For example, the Urumqi municipal authorities are committed to reduce the carbon emission from city development while pursuing “Ecological Civilization”^45^. Previous studies assessed the impact by comparing the SOCD_ISA_ against the SOCD measured in urban green spaces (i.e., the SOCD_PSA_ in our study)^30^,^31^. However, our study showed the converted green space areas accounted for less than 12% of the ISA expansion in Urumqi (Fig. 2c). Considering the landscape heterogeneity and the distinct SOC densities among the various land-cover types (Supplementary Figure S2)^12^,^31^, spatially explicit datasets which show the distribution of carbon pools and reveal the locations of carbon sinks/sources are necessary for effective carbon management^46^. Our study revealed a large amount of SOC lost in northwestern Urumqi, primarily because of the large-scale conversion of cropland to ISA in the area (Fig. 2d). Therefore, we recommend the municipal government to limit northwestern urban expansion and reduce the ISA in the newly developed land in this area. We also noticed two large downtown parks located at the southwestern and mid-western Urumqi shrunk dramatically from 1990 to 2010 (Fig. 2c). The conversions of these green spaces to ISA not only directly released 27.6 tons of SOC, but also could enhance the urban heat island effect that stimulates energy-related carbon emissions in Urumqi^47^.

### Uncertainties and future works

**Figure 3:**
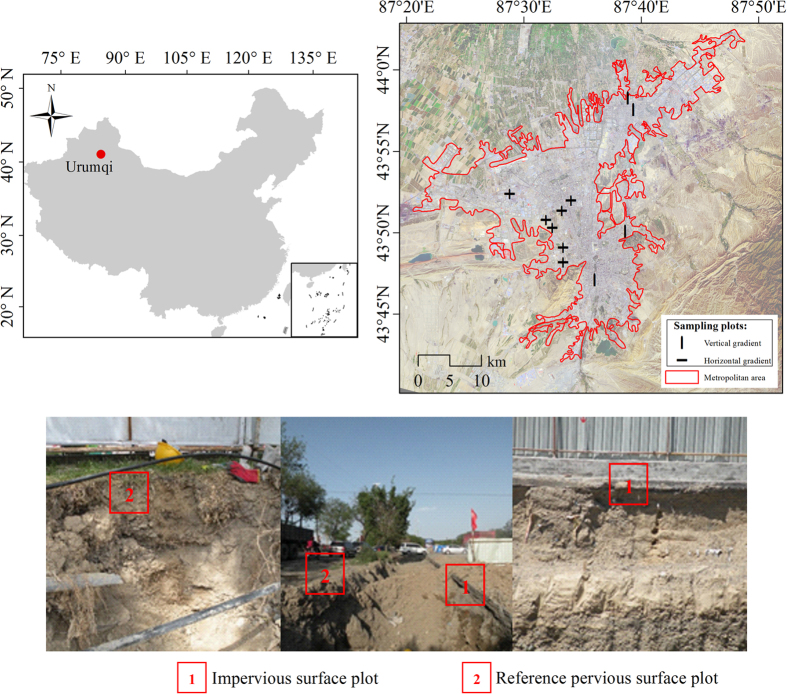
Location of the study area (the Urumqi metropolis, Xinjiang, China) and sampling sites with photos showing the soil profiles of the impervious surface (red box 1) and the adjacent pervious surface (red box 2) in a site. This map was generated by the one of the authors (Yan Yan) by using the ArcMap (version 10.2). The photographs in this map was taken by one of the authors (Yan Yan).

Our literature review found only three field investigations on the SOCD_ISA_, all of which studies SOCD_ISA_ in humid regions (Table 2). To our knowledge, this study was the first observational study on the SOC_ISA_ (and BD_ISA_) of a dryland city. We recognize that it is difficult to use 11 excavation sites for a 300–400 km^2^ city, and carefully selected the sites to ensure their representativeness. Compared to previous studies that focused on a few neighborhoods^30^,^34^,^48^, our sites were scattered across the city (Fig. 3) and represented the land-cover and land-use types in the city (see Methods and the supplementary Table S2). To improve the statistical confidence and reduce the uncertainties in urban soil research, SOC_ISA_ observations from different cities in various regions are essential, especially in the tropical and cold-zone, since all current studies were conducted in the temperate cities (Table 2).

## Methods

### Study area

Located at the northern slope of the Tianshan Mountains and on the southern edge of the Gurbantunggut Desert, Urumqi City (43.80° N, 87.60° E) is the economic and political center of the Northern-Tianshan Urban Agglomeration in Xinjiang province, China (Fig. 3)^49^. The city has a metropolitan area of 339 km^2^, and a population of 3 million^50^. Its built-up areas have the highest growth rate (7.4% per year) among all cities in Xinjiang^51^. With annual precipitation of 200–300 mm and a mean temperature of approximately 7.4 °C, the region represents the typical arid climate of northwestern China^51^. The regional landscape consists of agricultural oases and desert shrubland. Exotic broadleaf trees and turfgrass can also be found in the city. The main soil types include solonetzs and castanozems. The city displays poor soil development, which results in a shallow soil depth of 0–80 cm.

### Soil pit sampling method

With the help of a construction company, **s**oil samples under impervious surfaces were collected from 11 excavation sites (the profile pits were deeper than 80 cm) across Urumqi in an urban reconstruction project in March 2013 (Fig. 3). The sampling sites were selected carefully based on land type, soil type, and spatial distribution, to ensure their representativeness. The sealing of the impervious soils and the land-cover/vegetation types of the paired PSA plots (see below) had been stable for over 14 years (supplementary Figure S3). A detailed description of the site information is found in the supplementary Table S2. Each of the ISA sampling plots was paired with a PSA sampling plot from the adjacent open area, which was sampled in the same day. With permission from the landowner, a profile pit approximately 80~90 cm deep was dug in each plot. Using 100-ml sample rings, soil profiles in the pits were sampled at 20-cm intervals to an 80-cm depth where bedrock or alluvial grid gravel is encountered. We measured the BD and SOCC in each soil layer, then calculated the SOCD. All the soil samples were air-dried and sieved at 0.15 mm. The soil’s BD was measured using the volumetric ring method^52^. The SOCC was measured using the Mebius method involving Walkley-Black acid digestion^53^,^54^. Then, the SOCD was calculated by multiplying the SOCC by the soil BD. For each soil layer, the SOCD and BD values of the three sampling plots in a site were averaged to derive the site-level mean SOCD_ISA_, BD_ISA_, SOCD_PSA_, and BD_PSA_. We also sampled the horizontal pattern of SOCD_ISA_ and BD_ISA_ in 7 of the 11 sampling sites. Soil samples (0–80 cm depth) were taken at 0.5 m, 1 m, and 1.5 m from the edge of the ISA to the center.

### Mapping ISA in 1990 and 2010 from Landsat TM Imagery

The boundary of the Urumqi metropolitan area was defined according to the 1:1 000 000 land use/cover map of China (Fig. 2)^51^. Cloud free Landsat TM images acquired September 1990 and August 2010, which covered the metropolitan area of Urumqi (path/row: 143/30), were selected to classify five major land-cover types: water, ISA, green space, cropland, and remnant desert/bare ground in urban areas. After geometric rectification and radiometric calibration that transformed the original digital signals from the sensor to reflectance values, an improved image-based dark-object subtraction model was applied to perform atmospheric correction^55^,^56^. A linear spectral mixture analysis (LSMA) model was applied to extract the impervious surfaces (Supplementary Figure S4)^3^,^57^,^58^,^59^,^60^. To identify the major land-cover types effectively, the Landsat multispectral images were transformed into new components using minimum noise fraction transform; thus, most information can be found in the first three components^61^. From the scatterplots of the first three components, four endmembers-vegetation, soil, high-albedo object, and low-albedo object were identified^62^,^63^. Then, a constrained least square solution was applied to unmix the Landsat multispectral image into fraction images of these four endmembers^63^. Similar to Lu and Weng^41^, our visual checks and ground truth investigations indicated that the vegetation component represented the green spaces and croplands, the soil component represented the remnant desert/bare grounds, and the high- and low-albedo objects represented the ISAs and water bodies in the Urumqi metropolitan area.

Next, a decision tree classifier was used to develop the land-cover thematic maps of Urumqi from the four LSMA-derived fraction images. Supplementary Figure S4 illustrates the procedure of using the combination of the decision tree classifier and post processing method used to perform the urban land cover classification in this study. The training samples were selected through visual interpretation of 0.3 m resolution Worldview Images acquired in 2010 and 0.25 m resolution aerial photos acquired in 1990 over Urumqi. We first classified the selected images into water, ISA, remnant desert/bare ground, green spaces and cropland. Next, the 1:1 000 000 land use/cover map^51^ of Urumqi was used to separate the cropland from the urban green spaces. Stratified random sampling was conducted to assess the classification accuracy. A total of 200 points were randomly selected from the Worldview Images and the aerial photos. Producer’s accuracy, user’s accuracy, overall accuracy and the Kappa coefficient were calculated based on the confusion matrix (supplementary Table S3). The overall accuracy of classification of land use/cover in 1990 was 79%, and the Kappa coefficient was 0.68. The overall accuracy of classification in 2010 was 87%, and the Kappa coefficient was 0.72. The assessment indicated that the 30 m × 30 m resolution land-cover maps of Urumqi had reasonable accuracy for the follow-up study on ISA expansion impacts.

### Assessing the impacts of ISA expansion from 1990–2010 on the SOC stock of the Urumqi metropolitan area

The impacts of ISA expansion from 1990–2010 on the soil carbon stock of the Urumqi metropolitan area were estimated based on the land-cover maps of the two periods and the SOCD of each land-cover type (Fig. 2a–c). The mean SOCD underneath the impervious surfaces in Urumqi was represented by the mean SOCD_ISA_ values from the 11 sampling sites. Because all of our PSA sampling plots were located in the urban green spaces (including lawns and grassy medians between the sidewalk and the street), we approximated the SOCD of the green spaces with the SOCD_PSA_. Our field surveys indicated that most of the bare grounds in Urumqi were remnant deserts (shrub and scrubs). The mean organic carbon density of the desert/shrubland soils in the study region was 6.14 ± 0.88 kg C m^−2^^32^, and the SOCD of croplands (SOCD_crop_) was assigned the value of 9.94 ± 0.56 kg C m^−2^, which was the mean SOCD of the northern Tianshan croplands^32^. Because water bodies in Urumqi were generally deeper than 80 cm, their SOCD was set to 0 kg C m^−2^. This assumption would not significantly affect our results, because water bodies covered less than 1% of the dryland city, and accounted for less than 0.1% of the land-cover change. Finally, the 30 m × 30 m resolution SOCD maps of Urumqi in 1990 and 2010 were developed by assigning the mean SOCD value of each land-cover type to the corresponding pixels in the land-cover maps. The impact map of the ISA expansion from 1990–2010 was developed by comparing the SOCD map of 2010 to the SOCD map of 1990.

It should be noted that our sampling sites only represented roads, parking lots/squares, or traditional low-rise buildings (<7 stories). Modern high-rise buildings in Urumqi usually have basements or underground parking lots that require the complete removal of soil during construction. The SOC_ISA_ of the modern high-rise buildings should be assigned a value of zero. Furthermore, preliminary field investigations indicated that the proportion of modern high-rise buildings in an ISA was related to the land-use type, with transportation land having much fewer high-rise buildings than the urban residential areas (supplementary Table S4). Therefore, we first developed a land-use map of Urumqi from high-resolution aerial photos and the Worldview Images by visual interpretation and expert knowledge. Then, for each of the resulting 11 land-use types (supplementary Table S4), 30 samples were randomly selected. Visual interpretation and field investigation were conducted to identify the proportion of ISAs whose soils were completely removed (e.g., as a result of the construction of underground parking lots and basements). For land-use type *i*, the mean SOCD_ISA,*i*_ was calculated as equation (1):


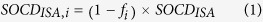


where *f*_*i*_ is the proportion of ISA in which soils were completely removed for land-use type *i* (supplementary Table S4); and SOCD_ISA_ is the mean SOCD_ISA_ of the 11 sampling sites. Then, by overlaying the land-use map to the land-cover map, we calculated the SOCD_ISA,*i*_ value for each ISA pixel in the land-cover map using equation (1).

## Additional Information

**How to cite this article**: Yan, Y. *et al.* Impacts of impervious surface expansion on soil organic carbon– a spatially explicit study. *Sci. Rep.* **5**, 17905; doi: 10.1038/srep17905 (2015).

## Supplementary Material

Supplementary Information

## Figures and Tables

**Table 1:** The observed soil bulk densities (BD), soil organic carbon content (SOCC), and derived soil organic carbon density (SOCD) of the impervious surface areas (ISA) and pervious surface areas (PSA) in Urumqi, Xinjiang, China.

Land caver	Values	BD ± S.E. (g cm^−3^)	SOCC ± S.E.(g kg^−1^)	SOCD ± S.E. (kg C m^−2^)
ISA	Means	1.62 ± 0.04	3.55 ± 0.36	5.36 ± 0.51
	Ranges	1.40–1.78	1.23–5.48	2.15–8.03
PSA	Means	1.49 ± 0.02	5.47 ± 0.58	8.08 ± 0.82
	Ranges	1.39–1.67	1.64–8.42	2.40–11.72
ISA-PSA		0.13 ± 0.04**	−2.12 ± 0.67**	−2.71 ± 0.9**

**means significant at *p < *0.01.

**Table 2:** Comparing the findings in this study with the previous studies.

Study Area	Climate regime	Sample size	Soil profile (cm)	SOCD_ISA_(kg C m^−2^)	Reference
			0–15	2.29	Raciti *et al.*^30^
Bronx & Brooklyn, New York, US	Humid temperate	62	45–60	0.61	
Leicester, UK	Humid temperate	17	various (from 7 to 115)	6.7–13.5	Edmondson *et al.*^12^
Nanjing, China	Humid temperate	14	0–20	2.35	Wei *et al.*^31^
Urumqi, China	Arid/semi-arid temperate	11 (with vertical gradient from surface)	0–20	1.71 ± 0.2	This study
			20–40	1.43 ± 0.22	
			40–60	1.30 ± 0.15	
			60–80	0.85 ± 0.16	
			0–80	5.36 ± 0.51	
		7 (with horizontal gradient from the edge of ISA)	0–80 (0.5 m from the edge)	5.44 ± 0.79	
			0–80 (1.0 m from the edge)	4.68 ± 0.69	
			0–80 (1.5 m from the edge)	3.91 ± 0.59	
Summary	All study areas located in the temperate zones in North Hemisphere.	104	Only the Urumqi study sampled the vertical and horizontal distribution of ISA soils systematically.	0.61–13.5, depending on the city location, and the depth and thickness of soil samples.	Including this study, so far there are only four field studies that reported observations of SOCD_ISA_.

Note: ISA-impervious surface area; SOCD-soil organic carbon density.
