# Supplementary material for: Impacts of impervious surface expansion on soil organic carbon – a spatially explicit study
Source: Sci Rep. 2015 Dec 8;5:17905. doi: 10.1038/srep17905 (PMC4672273; doi:10.1038/srep17905)
Supplement: Supplementary Information [file srep17905-s1.pdf]

Supplementary information for

Impacts of impervious surface expansion on soil organic carbon – a spatially explicit study

Yan Yan[1, 2], Wenhui Kuang[3], Chi Zhang[1, 4*], Chunbo Chen[1, 2]

1: State Key Laboratory of Desert and Oasis Ecology, Xinjiang Institute of Ecology and Geography, Chinese Academy of Sciences, Urumqi 830011, Xinjiang, China

2: University of Chinese Academy of Sciences, Beijing, 100049, China

3: Institute of Geographic Sciences and Natural Resources Research, Chinese Academy of Sciences, Beijing, 100101, China

4: Global Institute of Sustainability, Arizona State University, AZ, United States

* Corresponding author

Chi Zhang

Tel: (+86) 18690321105

Email: zc@ms.xjb.ac.cn

Xinjiang Institute of ecology and geography,

Chinese Academy of Sciences

Table of Content

List of Tables

Table S1. A list of SOC_{PSA} in cities based on literature review and this study .... SI 1
Table S2. Description of the sampling sites, and the observed BD and SOC in the ISA and PSA at 0-80 cm depth .... SI 2
Table S3. Classification accuracy assessments .... SI 3
Table S4. Estimating the mean soil organic carbon density (SOCD; kg C m^{-2}) of impervious surfaces in different land-use types after excluding the proportion of the impervious surface areas (ISA) in which soils were completely removed. .... SI 4

List of Figures

Figure S1. Comparison of the mean soil organic carbon densities of different land-cover types in the study region based on field observations and literature review. .... SI 5
Figure S2. Comparing the vertical patterns of SOC among the ISA, PSA, and rural land-uses such as cropland and desert (Wang et al., 2013). .... SI 6
Figure S3. Photos showing the land-cover types of the pervious areas and the sealing of the impervious soils had a history of over 14 years. Detailed descriptions for each site are found in the Supplementary Table S2. These photographs were developed by Chi Zhang using multiple high-resolution remote sensing data (e.g., aerial photos) from 2010-2013. .... SI 7
Figure S4. Framework showing the major steps for mapping urban land-cover distribution based on Landsat TM images ....SI 11

Table S1. A list of SOC_{PSA} in cities based on literature review and this study

City	Depth (cm)	Other cities	Unit	Reference
Hang Zhou china	0-100	18.6	kg C m^{-2}	Zhang and Zhou [1]
Hong Kong, China	0-100	4.2	kg C m^{-2}	Pouyat et al. [2]
Beijing, china	0-100	7.03	kg C m^{-2}	Wang [3]
Shanghai, china	0-100	10.55	kg C m^{-2}	Shi et al. [4]
Xuzhou, china	0-100	6.25±3.75	kg C m^{-2}	Si, 2013 [5]
New York, USA	0-100	2.9 ~ 28.5	kg C m^{-2}	Pouyat et al. [6]
Washington, USA	0-100	1.5	kg C m^{-2}	Pouyat et al. [2]
Baltimore, USA	0-100	9.9 ~ 12.2	kg C m^{-2}	Pouyat et al. [2]
Moscow, Russia	0-100	14.6	kg C m^{-2}	Pouyat et al. [2]
Chicago, USA	0-100	16.3	kg C m^{-2}	Pouyat et al. [2]
Leicester, UK	0-100	17.6	kg C m^{-2}	Edmondson et al. [7]
Kaifeng, China	0-100	6.99 ~ 11.02	kg C m^{-2}	Sun et al. [8]
Seoul, Korea	0-100	1.29 ~ 7.78	kg C m^{-2}	Bea et al., [9]
Leicester, UK	0-80	13.3 ~ 22.8	kg C m^{-2}	Edmondson et al. [10]
Guangzhou, china	0-60	6.86 ~ 10.72	kg C m^{-2}	Li [11]
Chicago, USA	0-60	4.3	kg C m^{-2}	Jo et al. [12]
Chunchuan, Korea	0-60	2.48	kg C m^{-2}	Jo, 2002 [13]
Stuttgart, Germany	0-30	3.1 ~ 23.2	kg C m^{-2}	Lorenz et al. [14]
Shanghai, China	0-30	11.2	g kg^{-1}	Hao et al. [15]
Nanjing, China	0-20	4.54	kg C m^{-2}	Wei et al. [16]
Beijing, China	0-20	3.07	kg C m^{-2}	Luo et al. [17]
Shanghai, china	0-20	3.93	kg C m^{-2}	Xu et al. [18]
Chongqing, China	0-20	2.0 ~ 3.7	kg C m^{-2}	Liu et al. [19]
Coventry, Stoke-on-Trent and Glasgow, UK	0-15	6.8 ~ 127.4	g kg^{-1}	Rawlins et al. [20]
Urumqi, China	0-80	8.08 ± 0.82	kg C m^{-2}	This study
Urumqi, China	0-60	5.76 ± 0.58	kg C m^{-2}	This study
Urumqi, China	0-20	2.30 ± 0.25	kg C m^{-2}	This study

SI 2

Table S2. Description of the sampling sites, and the observed BD and SOC in the ISA and PSA at 0-80 cm depth

Site ID	Longitude (DD)	Latitude (DD)	Elevation (m)	Soil type	Land-use type	ISA type	PSA type	ISA			PSA		
								BD_{ISA} (g cm^{-3})	$SOCC_{ISA}$ (g kg^{-1})	$SOCD_{ISA}$ (kg C m^{-2})	BD_{PSA} (g cm^{-3})	$SOCC_{ISA}$ (g kg^{-1})	$SOCD_{PSA}$ (kg C m^{-2})
1	87.65	43.84	826	Solonetzs	Industrial	Street	Street tree	1.51	3.81	5.75	1.42	6.96	9.90
2	87.48	43.87	725	Solonetzs	Industrial	Street	Street tree	1.63	2.24	3.66	1.46	1.64	2.40
3	87.56	43.86	769	Solonetzs	Institutional	Street	Street tree	1.75	1.23	2.15	1.42	7.06	10.00
4	87.53	43.86	797	Castanozems	Commercial	Parking lot	Urban woodland	1.65	3.69	6.10	1.49	5.47	8.06
5	87.54	43.84	816	Castanozems	Transportation	Highway	Urban lawn	1.68	4.76	8.03	1.67	6.18	10.30
6	87.56	43.82	853	Castanozems	Industrial	Street	Street tree	1.78	2.49	4.44	1.49	5.47	8.12
7	87.56	43.80	879	Castanozems	Transportation	Highway	Urban lawn	1.76	3.10	5.44	1.49	5.47	8.08
8	87.60	43.79	887	Unavailable	Commercial	Street	Street tree	1.60	3.29	5.28	1.62	2.50	4.03
9	87.57	43.87	765	Solonetzs	Commercial	Street	Street tree	1.40	5.48	7.64	1.50	5.54	8.29
10	87.64	43.97	591	Solonetzs	Residential	Paved backyard	Residential green space	1.61	2.70	4.35	1.39	8.42	11.72
11	87.66	43.96	613	Solonetzs	Residential	paved backyard	Residential green space	1.49	4.13	6.16	1.45	5.48	7.97

Table S3. Classification accuracy assessments

Year	Classified Data	Reference Data: Water	ISA	Urban GS	Cropland	Bare ground	Ref. totals	Class. Totals	Number Correct	PA	UA
1990	Water	2	0	0	0	0	2	2	2	100%	100.0%
	ISA	0	131	4	0	3	136	138	131	96.3%	94.9%
	Urban GS	0	1	12	0	2	19	15	12	63.2%	80.0%
	Cropland	0	0	0	10	0	16	10	10	62.5%	100.0%
	Bare ground	0	4	3	6	22	27	35	22	81.5%	62.9%
	Overall Classification Accuracy= 79% (i.e., 158/200), Overall Kappa Statistics = 0.6786										
2010	Water	2	1	0	0	0	3	3	2	66.7%	66.7%
	ISA	1	82	0	1	5	100	89	82	82.0%	92.1%
	Urban GS	0	4	18	4	2	20	28	18	90.0%	64.3%
	Cropland	0	1	0	11	0	18	12	11	61.1%	91.7%
	Bare ground	0	12	2	2	52	59	68	52	88.1%	76.5%
	Overall Classification Accuracy= 86.5% (i.e., 173/200), Overall Kappa Statistics = 0.7156										

Abbreviations: Urban GS - urban greenspaces; Ref. - reference; Class. - Classified; PA - producer’s accuracy; UA - user’s accuracy.

Table S4. Estimating the mean soil organic carbon density (SOCD; kg C m^{-2}) of impervious surfaces in different land-use types after excluding the proportion of the impervious surface areas (ISA) in which soils were completely removed.

Land-use type	Proportion of ISA, in which soil is completely removed (i.e., f_i in the equation 1)*	Mean SOCD (KgC m^{-2})
Commercial land	27%	4.06±1.31
Industrial land	6%	2.00±1.19
Urban residential area	54%	3.01±0.81
Urban village	~0%	3.31±1.56
Public service/ Institutional land	30%	3.51±0.59
Transportation	~0%	4.14±1.31
Remnant desert	~0%	0.34±0.31
Parks	~0%	0.40±0.55
Other urban greenspaces	~0%	2.91±1.28
Vacant/Construction	32%	0.01±0.02
Agricultural land	~0%	0.66±1.65
Overall	17%	2.31±1.55

* We found the soils under the high-rise buildings (> 7 stories) in Urumqi were usually completely removed in construction of basements or underground parking lots. The proportion of these high-rise built-up areas to the total ISA of a land-use type was derived by field investigations and visual classification (based on high-resolution satellite and aerial photos).

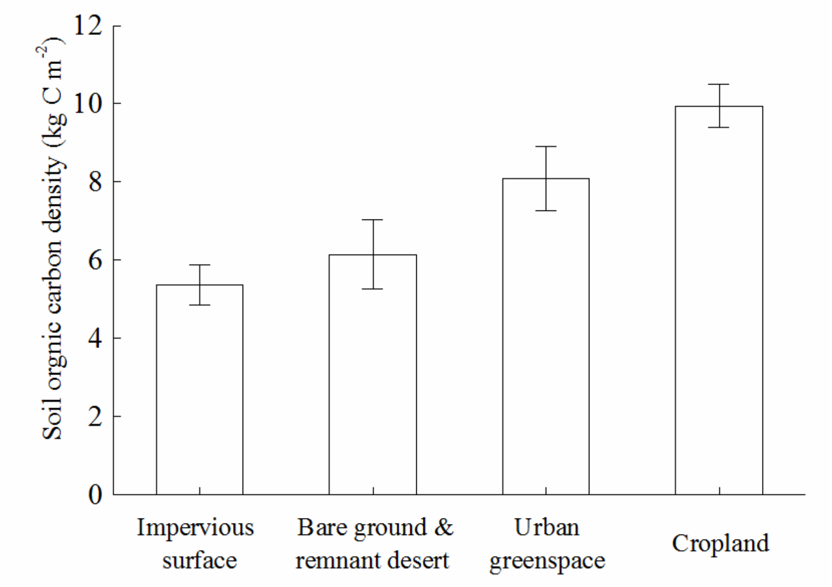

Figure S1. Comparison of the mean soil organic carbon densities of different land-cover types in the study region based on field observations and literature review.

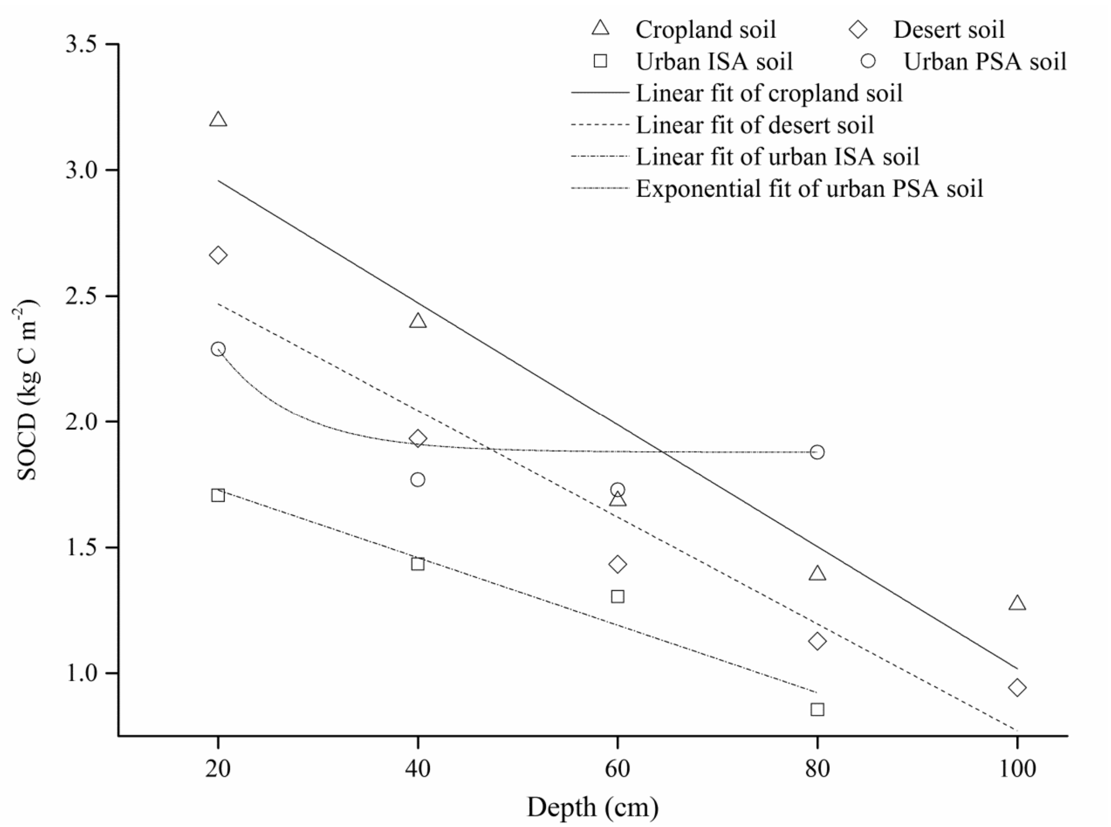

Figure S2. Comparing the vertical patterns of SOC among the ISA, PSA, and rural land-uses such as cropland and desert (Wang et al., 2013).

SI 7

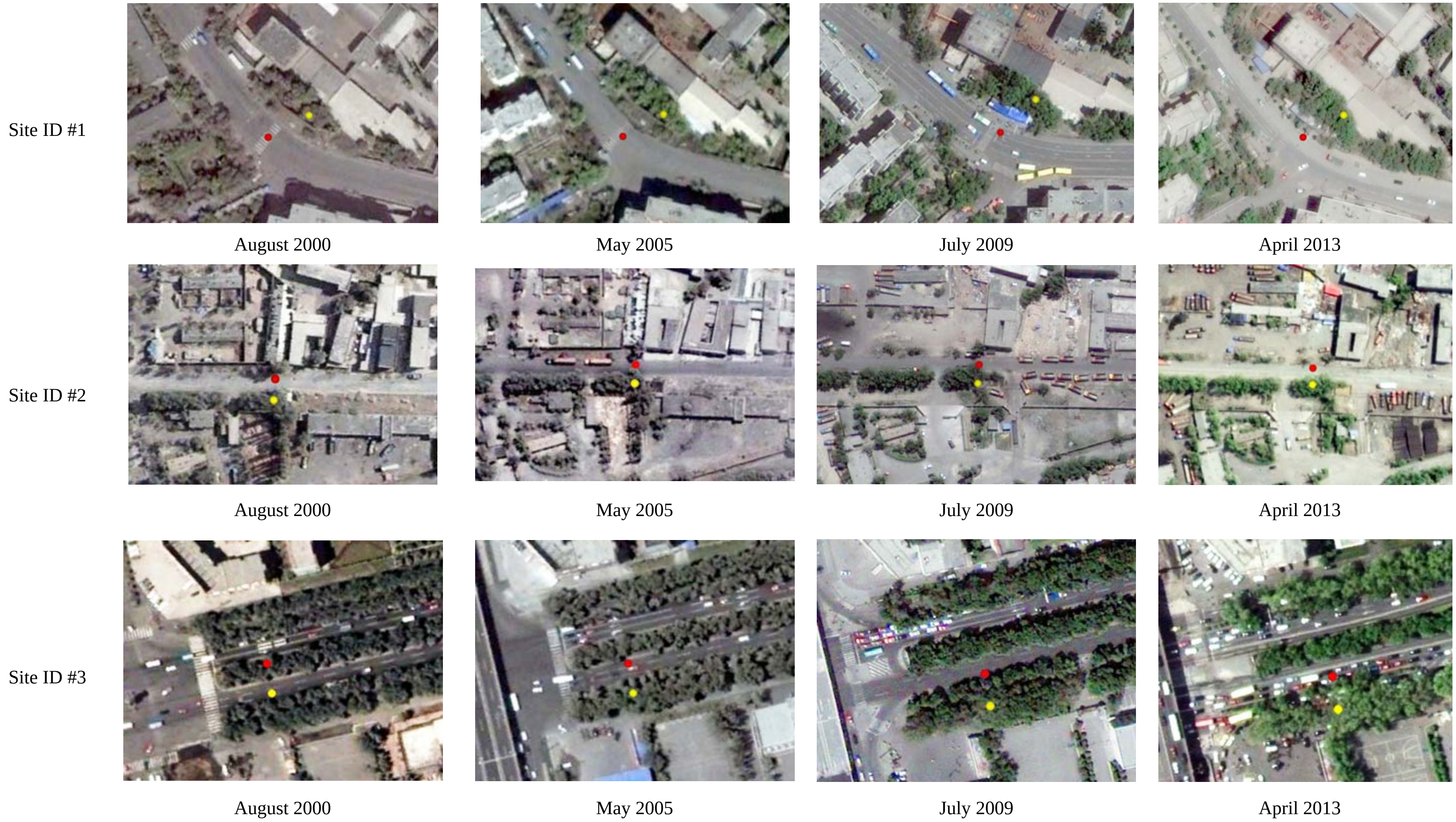
Site ID #1
August 2000
May 2005
July 2009
April 2013
Site ID #2
August 2000
May 2005
July 2009
April 2013
Site ID #3
August 2000
May 2005
July 2009
April 2013

SI 8

Site ID # 4

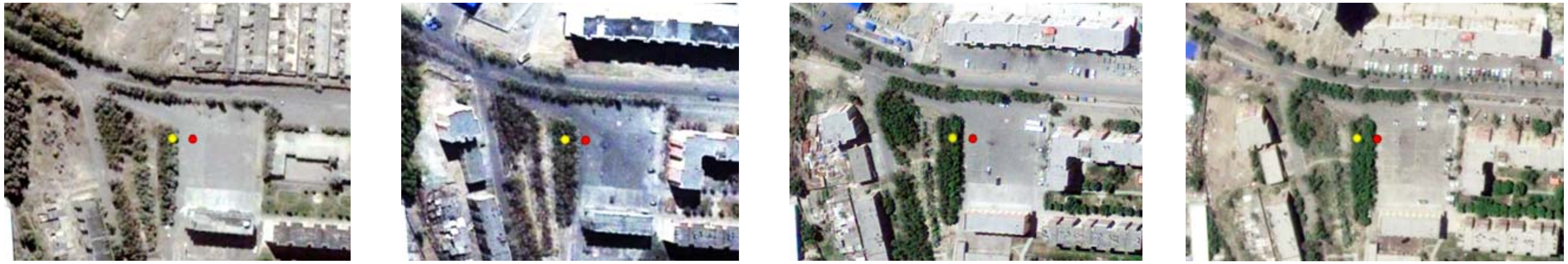

August 2000 | May 2005 | July 2009 | April 2013

Site ID # 5

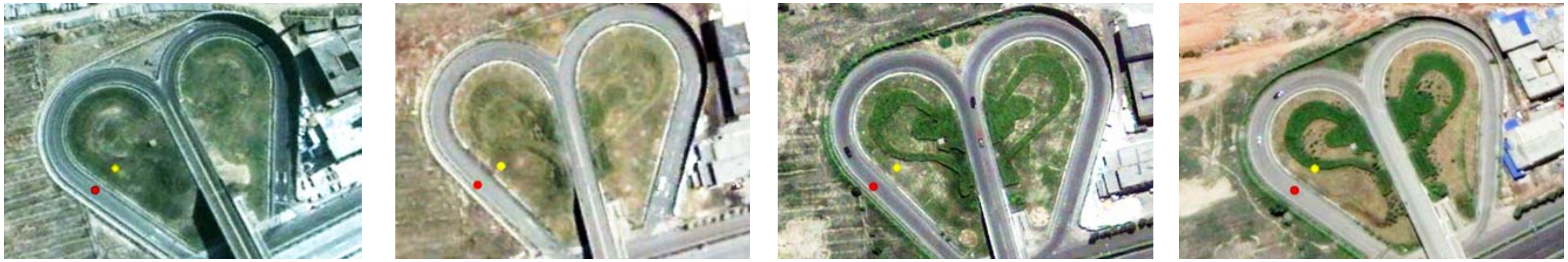

August 2000 | May 2005 | July 2009 | April 2013

Site ID # 6

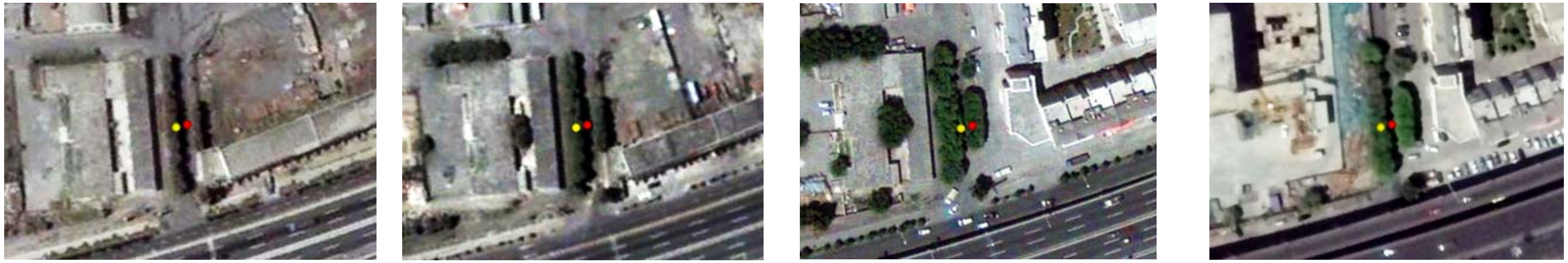

August 2000 | May 2005 | July 2009 | April 2013

SI 9

Site ID # 7

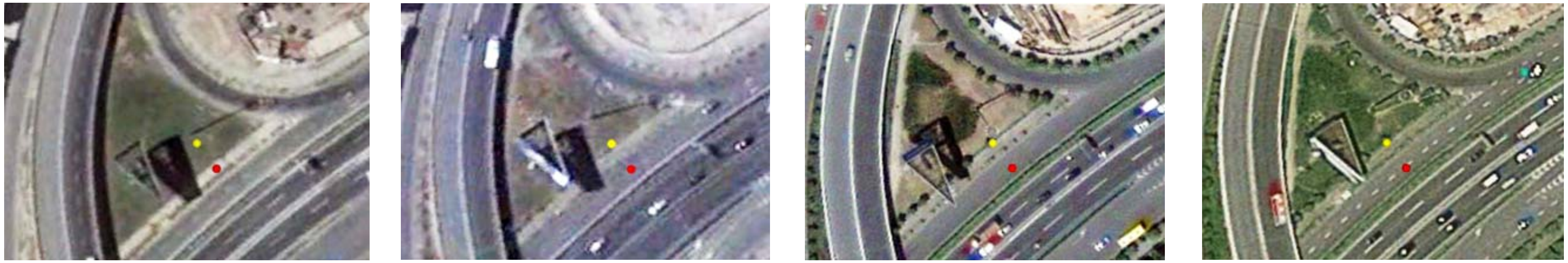

August 2000 | May 2005 | July 2009 | April 2013

Site ID # 8

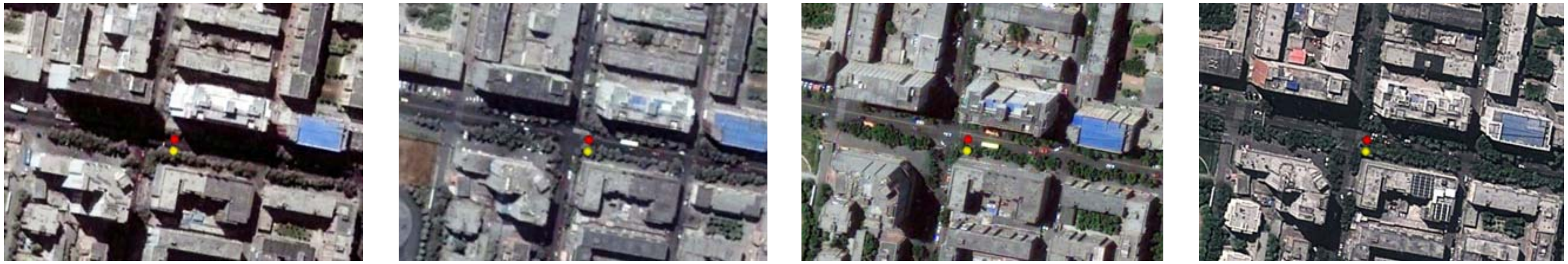

August 2000 | May 2005 | July 2009 | April 2013

Site ID # 9

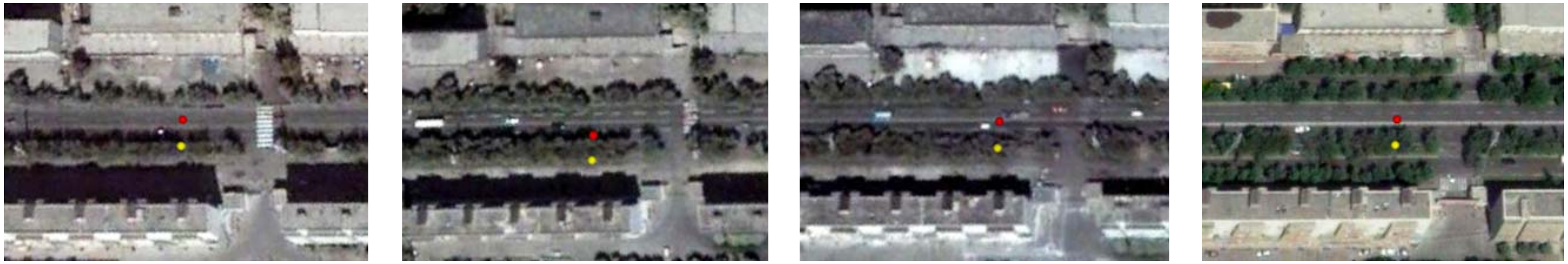

August 2000 | May 2005 | July 2009 | April 2013

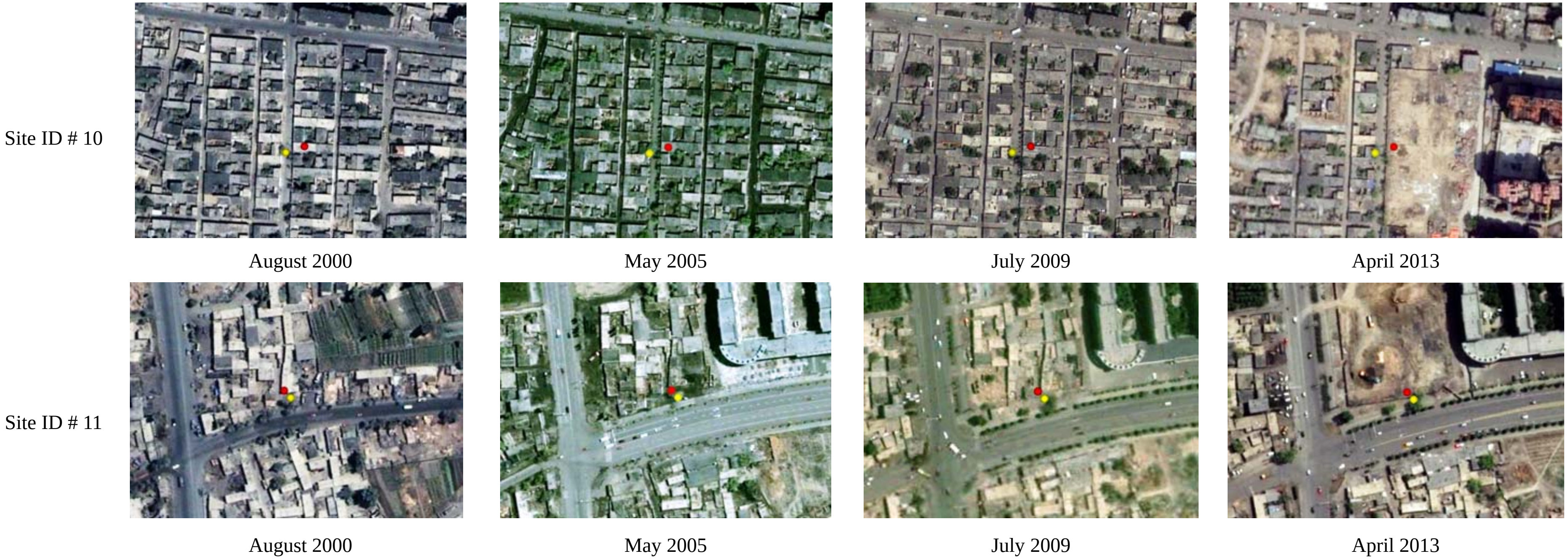

Figure S3. Photos showing the land-cover types of the pervious areas and the sealing of the impervious soils had a history of over 14 years. Detailed descriptions for each site are found in the Supplementary Table S2. These photographs were developed by Chi Zhang using multiple high-resolution remote sensing data (e.g., aerial photos) from 2010-2013.

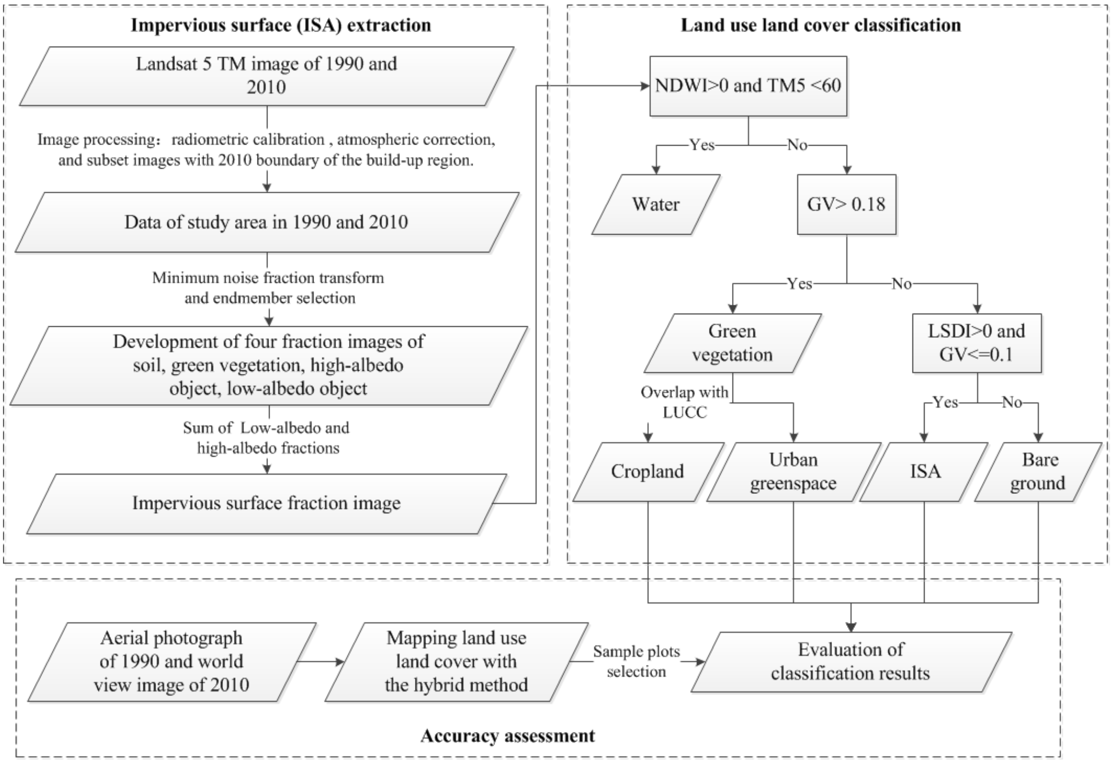

Figure S4. Framework showing the major steps for mapping urban land-cover distribution based on Landsat TM images

References

1 Kui, Z. M. & Cui, z. Characterization of organic matter accumulated in urban soils in the Hangzhou City. *Chinese Journal of Soil Science* 37, 19-21 (2006).

2 Pouyat, R. V., Yesilonis, I. D. & Nowak, D. J. Carbon storage by urban soils in the United States. *J Environ Qual* 35, 1566-1575, doi:10.2134/Jeq2005.0215 (2006).

3 Wang, D. S. *studies on net carbon reserves in Beijing urban landscape green based on biomass measurement* PhD thesis, Beijing Forestry University, (2009).

4 Shi, L. J., Zheng, L. B., Mei, X. Y., Yu, L. Z. & Jia, Z. C. Characteristics of soil organic carbon and total nitrogen under different land use types in Shanghai. *Chinese Journal of Applied Ecology* 21, 2279-2287 (2010).

5 Si, Z. G., Cao, Y. C. & Yu, Y. C. Research progress on the estimation of urban soil organic carbon storage. *Ecological Economy* 2, 103-105 (2011).

6 Pouyat, R., Groffman, P., Yesilonis, I. & Hernandez, L. Soil carbon pools and fluxes in urban ecosystems. *Environmental Pollution* 116, Supplement 1, S107-S118, doi:http://dx.doi.org/10.1016/S0269-7491(01)00263-9 (2002).

7 Edmondson, J. L., Davies, Z. G., McHugh, N., Gaston, K. J. & Leake, J. R. Organic carbon hidden in urban ecosystems. *Sci Rep-Uk* 2, doi: 10.1038/Srep00963 (2012).

8 Sun, Y. L., Ma, J. H. & Li, C. Content and densities of soil organic carbon in urban soil in different function districts of Kaifeng. *J Geogr Sci* 20, 148-156, doi:10.1007 / s11442-010-0148-3 (2010).

9 Bae, J. & Ryu, Y. Land use and land cover changes explain spatial and temporal variations of the soil organic carbon stocks in a constructed urban park. *Landscape Urban Plan* 136, 57-67, doi:DOI 10.1016/j.landurbplan.2014.11.015 (2015).

10 Edmondson, J. L. *et al.* Urban tree effects onsoil organic carbon. *Plos One* 9, doi: 10.1371/journal.pone.0101872 (2014).

11 Li, X. M. *The research on carbon circulation of forest ecosystem in Guangzhou city* Master of Scicence thesis, Central south university of forestry and technology, (2008).

12 Jo, H. K. & Mcpherson, E. G. Carbon storage and flux in urban residential greenspace. *J Environ Manage* 45, 109-133, doi:DOI 10.1006/jema.1995.0062 (1995).

13 Jo, H. K. Impacts of urban greenspace on offsetting carbon emissions for middle Korea. *J Environ Manage* 64, 115-126, doi:DOI 10.1006/jema.2001.0491 (2002).

14 Lorenz, K. & Lal, R. Biogeochemical C and N cycles in urban soils. *Environ Int* 35, 1-8, doi: 10.1016/j.envint.2008.05.006 (2009).

15 Hao, R. J., Fang, H. L. & Shen, L. Y. Distribution characteristics of soil organic carbon and total nitrogen in greenbelt soil in Shanghai center city. *Journal of Nanjing Forestry University (Natural Science Edition)* 35, 49-52 (2011).

16 Wei, Z. Q., Wu, S. H., Zhou, S. L., Li, J. T. & Zhao, Q. G. Soil organic carbon transformation and related properties in urban soil under impervious surfaces. *Pedosphere* 24, 56-64 (2014).

17 Luo, S. H., Mao, Q. Z. & Ma, K. M. Comparison on soil carbon stocks between urban and suburban topsoil in Beijing, China. *Chinese Geogr Sci* 24, 551-561, doi:DOI .1007/s11769-014-0709-y (2014).

18 Xu, N. Z., Liu, H. Y., Wei, F. & Zhu, Y. P. Urban expanding pattern and soil organic, inorganic carbon distribution in Shanghai, China. *Environ Earth Sci* 66, 1233-1238, doi:DOI 10.1007/s12665-011-1334-z (2012).

19 Liu, Y., Wang, C., Yue, W. Z. & Hu, Y. Y. Storage and density of soil organic carbon in urban topsoil of hilly cities: A case study of Chongqing Municipality of China. *Chinese Geogr Sci* 23, 26-34, doi:DOI 10.1007/s11769-013-0585-x (2013).

20 Rawlins, B. G. *et al.* Methods for estimating types of soil organic carbon and their application to surveys of UK urban areas. *Soil Use Manage* 24, 47-59, doi:DOI 10.1111/j.1475-2743.2007.00132.x (2008).

21 Wang, Y. G. et al. Vertical distribution of soil organic carbon in different land cover types in Northern Piedmont of the Tianshan Mountains. Arid Zone Research 30, 913-918 (2013).
